# Supplementary material for: Optimization and Validation of Efficient Models for Predicting Polythiophene Self-Assembly
Source: Polymers (Basel). 2018 Nov 26;10(12):1305. doi: 10.3390/polym10121305 (PMC6401914; doi:10.3390/polym10121305)
Supplement: Supplementary file 1 [file polymers-10-01305-s001.pdf]

Supplementary Materials: Optimization and Validation of Efficient Models for Predicting Polythiophene Self-Assembly

Evan D. Miller [1], Matthew L. Jones [1], Michael M. Henry[1], Paul Chery[2], Kyle Miller[3] and Eric Jankowski [1,*]

1. Force-Field Constraints for Bonded Atoms

Here we present the intra-molecular interactions, e.g. two-body bond, three-body angle, and four-body dihedral constraints governing our P3HT model. These bonded interactions are adapted from the atomistic models developed by Huang and Bhatta [1,2]. As we employ united-atom models in this work, we compare our force-field values with the OPLS-UA force-field to ensure that we are not introducing spurious interactions when adapting the atomistic model [3].

Bond potentials are harmonic:

$$E_{\text{bond}} = \frac{1}{2}k(l - l_0)^2, \quad (1)$$

in which k is the spring constant and l_0 is the equilibrium bond length. The k and l_0 values are presented in Table S1. It should be noted that the CA-CA bond parameters presented in Table S1 only apply to bonds between thiophene rings. The thiophene rings themselves are represented as rigid bodies, and so bonds within the rings do not change during the simulation. If a flexible model were to be used for the thiophene ring, then distinct bead types would be needed to distinguish bonds between and within rings.

Table S1. The bond constraints used in our force-field to simulate P3HT. †: Bonds between two monoers, rather than within a single, rigid thiophene. ‡: Bonds completely enclosed by a rigid body, which are therefore fixed at l_0.

Bond	l_0 (Å)	k (kcal mol^{-1} Å^{-2})
CA-CA†	1.43	392.0
CT-CT	1.53	268.0
CA-CT	1.51	300.0
CA-S‡	1.71	291.0

Angles constraints are also harmonic:

$$E_{\text{angle}} = \frac{1}{2}k(\theta - \theta_0)^2, \quad (2)$$

in which θ_0 is the equilibrium bond angle. The angle constraints are presented in Table S2. As before, the CA-CA-CA and CA-CA-S angles specified describe angle constraints between monomers, rather than those in the rigid thiophene ring.

The dihedral parameters are defined by a multi-harmonic function in the form:

$$E_{\text{dihedral}} = \sum_{n=0}^{4} k_n cos^n \varphi, \quad (3)$$

in which φ is the dihedral angle. The dihedral constraints are presented in Table S3.

Due to the rigid bodies used in this investigation, some dihedrals become ambiguously defined if the force-field from the literature are used. For instance, the CA-CA-CA-CT and the CA-CA-CA-CA dihedral constraints are already considered by the CA-CA-CT-CT and S-CA-CA-S dihedrals respectively. Furthermore, in the case of CA-CA-CA-CT, two possible definitions are

Table S2. The angle constraints used in our force-field to simulate P3HT. †: Angles between two monomers, rather than within a single, rigid thiophene. ‡: Angles completely enclosed by a rigid body, which are therefore fixed at θ_0.

Angle	θ (rad)	k (kcal mol^{-1} rad^{-2})
CA-CA-CA†	2.27	54.7
CA-CA-S†	2.09	41.7
CA-S-CA‡	1.62	86.0
CA-CT-CT	2.16	70.0
CA-CA-CT	2.15	70.0
CT-CT-CT	1.97	37.5

Table S3. The angle constraints used in our force-field to simulate P3HT. †: Dihedrals between two monomers, rather than within a single, rigid thiophene.

Dihedral	k_0 (kcal mol^{-1})	k_1	k_2	k_3	k_4
S-CA-CA-S	2.9533	0.1571	-4.2326	0.39979	1.8855
CA-CA-CA-S†	2.9533	-0.1571	-4.2326	-0.39979	1.8855
CA-CA-CT-CT	0.3175	1.127	14.143	-22.297	6.7188
CA-CT-CT-CT	2.4469	-6.3946	10.747	30.695	11.139
CT-CT-CT-CT	1.8922	-3.4904	1.4665	7.1418	0.2859

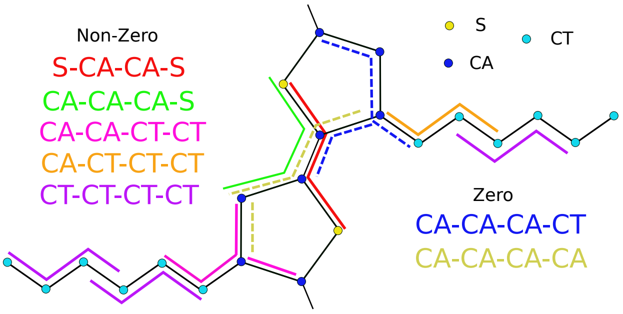

Figure S1. The flexible dihedrals used in this investigation that are not completely enclosed by a rigid body. Reducing the number of distinct atom types in this model (compared to the all-atom models from which they are derived) leads to some conflicts in the dihedral constraints. To avoid this, the coefficients for some of these dihedrals (CA-CA-CA-CT and CA-CA-CA-CA) are set to zero.

applicable depending on whether the CA is located in the same ring as the alkyl sidechain or not. When the CA is in a neighboring ring to the monomer containing the CT, the dihedral is in the cis conformation, however, when CA is in the same ring as the specified side chain, the dihedral is in the trans conformation. This ambiguity leads to instabilities in the system when the dihedral parameters are not set to 0. As such, we set the CA-CA-CA-CT and CA-CA-CA-CA dihedral coefficients to zero, effectively deactivating them in our force-field. Figure S1 shows which dihedral constraints are considered (solid lines) and which are omitted (dashed lines), to highlight the redundancies that exist due to reduction to three atom types and the incorporation of the rigid body. We therefore assume that the other carbon aromatic to chain (CA-CA-CT-CT and CA-CT-CT-CT) dihedrals are sufficient in describing the position of the chains relative to the rings and that the (S-CA-CA-S and CA-CA-CA-S) dihedrals are sufficient in describing the orientation of the thiophene rings along the backbone.

2. The Effect of Including Explicit Charges in the Model

Modeling the long-range electrostatic interactions between P3HT chains is both computationally expensive and challenging to do accurately. Including explicit partial charges results in a factor of 3 increase in simulation time for the systems studied here. Additionally, small conformational changes in conjugated polymers such as P3HT give rise to significant changes in electron densities and therefore the partial charges associated with each atom fluctuate over time and space. Furthermore, prior calculations for modeling P3HT electrostatics do not reach consensus on which partial charges are correct, or sufficient [2,4,5] In this section we compare our base case implicit charge model against one with explicit partial charges whose forces are calculated with the Fourier based particle-particle-particle-mesh Ewald summation method [6]. We quantify structural and performance differences between these approaches and show the implicit charge model sufficiently captures the relevant assembly physics.

The charges used in this study are determined through first-principle calculations with the NWChem software [7] with the Becke, three-parameter, Lee-Yang-Parr (B3YLP) hybrid functional [8] with the 6-311++g** basis set [9]. The partial charges for the atoms within the thiophene ring from literature and those calculated in this study are shown in Table S4. From the values presented in

Table S4. The charges for atoms given in literature for all-atom simulations. *Indicates that this was a united-atom simulation. †Indicates that these were calculated as part of this work by the NWChem program [7].

Study	S	C1	C2	C3	C4
Bhatta [2]	-0.22	0.18	-0.04	-0.31	0.03
Obata [10]	-0.07	0.12	-0.07	-0.27	0.05
Moreno [11]	-0.12	-0.01	-0.01	-0.18	-0.03
Huang [1]	-0.15	-0.14	0.075	-0.18	-0.18
Borzdun [12]	0.33	-0.24	-0.28	-0.16	0.08
D'Avino* [13]	-0.06	-0.21	0.22	0.10	-0.29
Trimer†	-0.17	0.28	-0.03	-0.37	-0.11
Pentamer†	-0.16	0.24	0.01	-0.37	-0.14

Table S4 we utilize the pentamer values for our simulations (Figure S3a). Further, we note that we also zero-out the charge in the simulation by subtracting the average per-particle charge from every simulation bead to account for rounding errors that may occur on individual atoms.

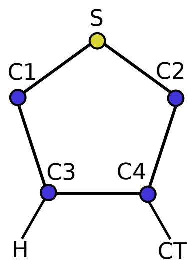

Figure S2. Labels for identifying the atoms in the thiophene in Table S4.

Since a united-atom model is used in this work, we sum the partial charges of the hydrogen atoms into the atom to which they are directly bonded. These values are shown in Table S5. The CTN labels, in which N=1→6, are used to identify the aliphatic carbons with CT1 being bonded to the thiophene ring and CT6 being the farthest away from the thiophene ring. We note that the C3 presented in Table S5 is the same as that shown in Table S4, but with the hydrogen considered. Because the works of Moreno and Huang consider polythiophenes with no alkyl sidechains, we exclude these works from the list of the side-chain charges.

Table S5. The charges from literature summed up so the hydrogens are considered with the carbons. C3 is the same as above. CT1 is bonded to the thiophene ring and each CT$N = 1 \rightarrow 6$ is extending from the ring.

Study	C3	CT1	CT2	CT3	CT4	CT5	CT6
Bhatta [2]	-0.08	0.14	-0.04	0.03	0.04	-0.03	-0.01
Obata [10]	-0.11	0.07	0.03	-0.04	0.01	0.09	-0.08
Borzdun [12]	0.06	0.02	-0.01	-0.01	0.0	0.0	0.0
D'Avino [13]	0.10	0.22	0.08	-0.07	0.01	0.04	-0.03
Trimer	-0.12	0.19	0.06	-0.16	0.11	0.07	-0.10
Pentamer	-0.13	0.19	0.07	-0.17	0.10	0.09	-0.10

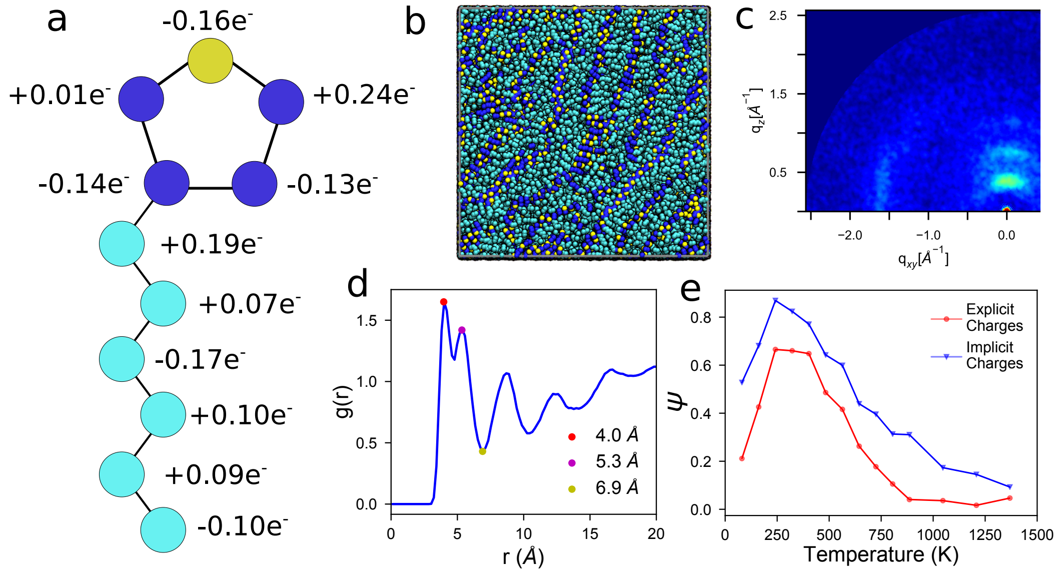

Figure S3. (a) The partial charges applied to the thiophene ring in the explicit-charge model. These charges were generated from a pentamer chain using first-principle calculations. Incorporating these into the equilibrated implicit-charge structure at the same state-point Figure 7a results in (b), which is visually indistinguishable (CA-dark blue, S-yellow, CT-cyan; state-point: explicit charges, $T \sim 600$ K, $\varepsilon_s = 0.8$, $\rho = 1.11$ g/cm^3). Short- and long-range structural features observed in the (c) GIXS (averaged over 6 orientations) and (d) thiophene centroid RDF (compared to Figure 6a and Figure 7e respectively) show that the explicit-charge model with $\sigma_{LJ} = 3.7$ Å is consistent with the implicit-charge model with a reduced $\sigma_{LJ} = 3.44$. (e) The explicit- (red circles) and implicit-charge (blue triangles) systems exhibit similar trends in order (ψ) over different temperatures (state-point: ε_s = 0.2 and ρ = 0.72 g/cm^3). However, explicit-charge systems systematically obtain a lower degree of order.

We assign partial charges to simulation beads (Figure S3a). We then employ scattering, RDF, and cluster analyis described above to to determine the differences in molecular packing. In our optimized implicit-charge model, we reduce the thiophene ring bead diameters to ~ 3.44 Å from the ~ 3.7 Å used in OPLS-UA and Amber [3,14], which permits the thiophene rings to π-stack at the same length-scale as in experiments. In the explicit charge model benchmarked here, we employ the partial charges, but do not modify the Lennard-Jones ϵ or σ parameters from our optimized implicit-charge model. These parameters *should* be updated to create an optimized explicit-charge model, but we hypothesize that such efforts are not worth their cost and check here to see how the addition of explicit charges affects structure and performance.

To test whether explicit charges destabilize an already-equilibrated structure from an implicit-charge model (Figure 7a), we instantaneously "turn on" electrostatics with the above partial charges and re-equilibrate a 100 15-mer $T = 600$ K, $\rho = 1.11$ g/cm^3 Protocol (2) system over 0.3 μs. The

resulting morphology (Figure S3b) is not significantly destabilized, and shows qualitative agreement with GIXS and RDF metrics of the implicit-charge case. The primary quantitative difference arises from the electrostatic repulsion of identical beads when thiophene rings are aligned: With explicit charges, aligned rings are on average 4.0 Å apart, instead of 3.9 Å as in the implicit charge case.

To test differences in self-assembled structure we perform new simulations at $\varepsilon_s = 0.2$ with Protocol (2) and the base-case 100 15-mers, but with explicit electrostatic charges active throughout. Figure S3e shows that order parameter ψ dependence on temperature is qualitatively the same for both implicit- and explicit-charge models. Independent of charge consideration, we observe kinetic trapping at low temperatures (< 250 K), increased order at moderate temperatures (250 K to 500 K), and decreasing order as temperatures increase (> 750 K). The explicit-charge model is systematically less ordered ($\psi_{explicit} \sim \psi_{implicit} + 0.1$) than the implicit model across all temperatures studied here, but otherwise assembles the same structures: lamellae of π-stacked backbones below a transition temperature and disordered melts above. On one hand, we interpret these results to justify the factor of 3 performance benefit of the implicit charge model. On the other hand, we acknowledge that the self-assembled structures are not identical between these two cases, and this *could* have significant ramifications, for example in predicting charge mobilities. We therefore recommend using implicit charge models for screening large parameter spaces as performed here, and then subsequently performing more expensive simulations when they are warranted for additional insight.

3. The Effect of Considering Longer Chains

In this section we compare performance and packing of "long" 50 monomer (50mer) chains relative to the 15mer base-case used throughout this work. Commercially available P3HT chains (from e.g. Sigma-Aldrich, Reike Metals) are significantly longer, typically ranging from 125 to 625 monomers, and more polydisperse (PDI$\sim$ 2) despite the feasibility of synthesizing narrow chain length distributions [15–17]. However, 50mers are the longest chains that we can simulate at the same conditions as the 15mers, while ensuring a chain cannot interact with itself through the periodic simulation boundaries. This is a conservative modeling choice, as we do not anticipate that such self-interactions would significantly impact self-assembled morphology. Additionally, we expect the 50mer chains to provide useful information about the effect of simulating longer chains compared to the 15mer base case, despite being shorter than commercially available. We simulate 300 50mer chains at $\rho = 1.11$ g/cm^3 using Protocol (2) at $T \sim 600$ K and $\varepsilon_s = 0.8$, and compare against 1000 15-mers at the same conditions as subsection 4.5 of the maintext.

The 50mer systems (Figure S4a) demonstrate less ordering than their 15mer counterparts under the same conditions. Using clustering analysis, we still see short-range order exists in the form of small crystallites that are randomly oriented with respect to each other (Figure S4b). The lack of long-range order for 50mers is apparent from GIXS (Figure S4c), which lacks distinct reflections corresponding to lamellae of *pi*-stacked backbones as seen before. The thiophene ring RDF (Figure S4d) shows some short-range ordering, with anti-aligned thiophene rings (expected peak at 5.3 Å) less common in 50mer systems. The major structural difference between 50mers and 15mers is that the 50mers are observed to "fold" and form π-stacks with themselves (Figure S4e) Such self-stacking allows a single chain to form multiple layers in one crystallite, and is observed experimentally [18–20].

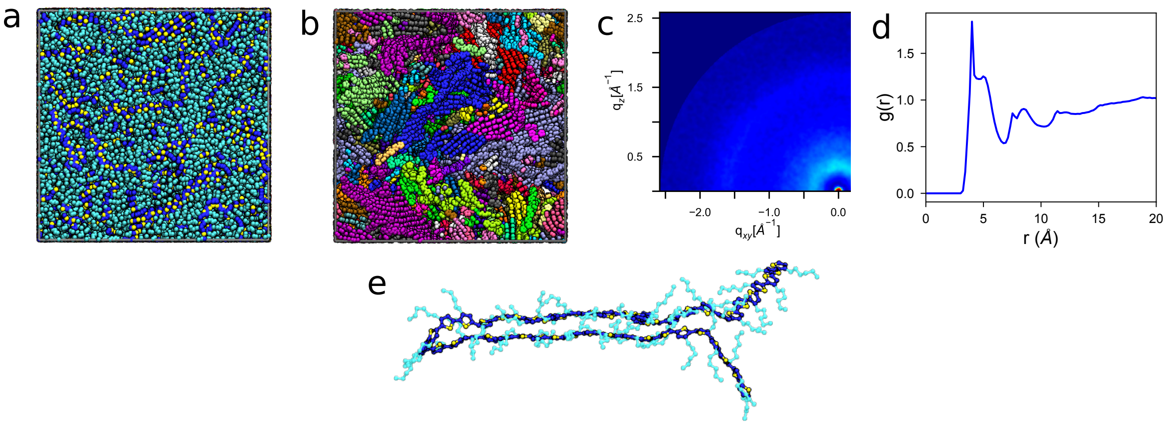

Figure S4. (a) 300 × 50mer chains produce morphologies containing no visually discernible periodic order at $T \sim 600$ K and ε_s = 0.8 (CA-dark blue, S-yellow, CT-cyan). (b) However, short-range order is visible when thiophene rings are colored by cluster. (c) Short-range order is confirmed through simulated GIXS (averaged over 60 orientations) and (d) the RDF of the thiophene centroids, which both show less defined peaks than in the 15mer case (Figure 6a and Figure 7e respectively). (e) The 50mer chains are long enough to undergo π-stacking with themselves, which is observed experimentally [18–20] and not observed with the shorter chains.

We estimate the time required for the system containing 300 × 50 monomer long chains (50mer) to relax to an equilibrated structure compared to the systems containing 1000, 15 monomers long chains (15mer) by comparing the evolution of the Lennard-Jones potential energies and also the structure factor over time. As a first test, we assume that the systems have the same equilibrated structure and that these two structures will have the same non-bonded energies. Therefore, we fit the 50mer and 15mer Lennard-Jones energy over time to equations and determine where the 50mer's equation will equal the final energy of the 15mer system. In choosing what equation should be used to represent

the energy evolution, we qualitatively observe that the P3HT systems undergoes three stages during relaxation: fast exponential decay, slow approximately linear decay and constant. As such, we fit the per-particle potential decay over simulation time t_s to:

$$f(t_s) = a \times exp(b \times t_s) + c \times t_s + d, \quad (4)$$

to get the coefficients a, b, c, and d for the exponential and linear terms; the fitted curve is showed by the dashed red line in Figure S5a. With the coefficients known, we solve the equation to determine when it would equal the final energy of the 15mer system, which results in approximately 9,000 times longer for the 50mer system to run to equal the final energy as the 15mer system.

However, it is not necessarily valid to assume that the 50 and 15mer systems will have the same final energy. For instance, it is possible that the 50 system (which is more prone to entanglements) will reach a metastable state and will require the lifetime of the universe to relax out of the metastable state. Such behavior often occurs in real systems, in that P3HT devices have crystalline and amorphous regions rather than the more energetically favored perfect crystal. As such, if we do not assume that the two systems will have the same equilibrium structure and hence same energy, we can instead utilize the rate of decay of the energy as our descriptor for system evolution. Because the linear decay is the slowest process, we can compare the ratio of the linear coefficients between the 50mer and 15mer system to describe the difference in relaxation times. As such, we find that the 50mer's coefficient is -1.94 $\times$ 10^{-5} and the 15mer's is -3.6 $\times$ 10^{-5}. This suggests a $\sim$ 2$\times$ faster decrease in energy in the 15mer system than the 50mer system.

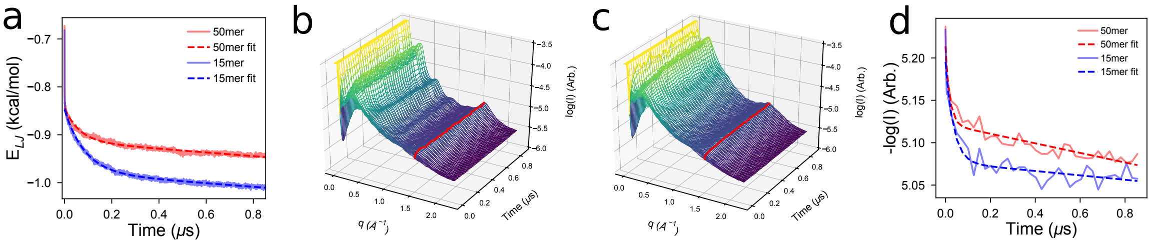

Figure S5. (a) The potential energy in the (blue) 15mer system decays faster and achieves a final lower per-particle than the (red) 50mer system. The structure factor - a radial average of the GIXS pattern - shows the growth of peaks corresponding to (red) π-stacking over time in the (b) 15mer and the (c) 50mer system. (d) The evolution of the red peaks in b and c show that the 15mer system reaches a stable state faster than the 50mer system.

Rather than using the energy, which is an indirect measure of the structure's evolution, we can instead utilize a direct measurement of the structure such as the structure factor. We therefore conduct the scattering experiment to obtain the structure factor - a radial average of the GIXS pattern - every 25 ns of simulation run time (Figure S5b and c). We record the height of the peak in the structure factor located at 1.6 Å^{-1}, corresponding to π-stacking, over time. We again fit the evolution of the structure factor peak over time to an exponential function and compare the relative changes in structure evolution between the 50mer and 15mer systems (Figure S5d). From this, we again predict that the 50mer system will require twice as long to reach equivalent scattering intensities of that of the 15mer system. Because the structure factor is the most direct calculation of structure and it agrees with the change of energy we conclude that the 50mer system requires twice as long as the 15mer system to order. In summary, the longest chains that can be practically equilibrated should be used, 15mers are sufficient for predicting experimental GIXS patterns, and systems of 50mers can in principle be equilibrated, but the factor of two increase to simulation time precludes routine sweeps of large parameter spaces.

4. Linking System Evolution to Energy

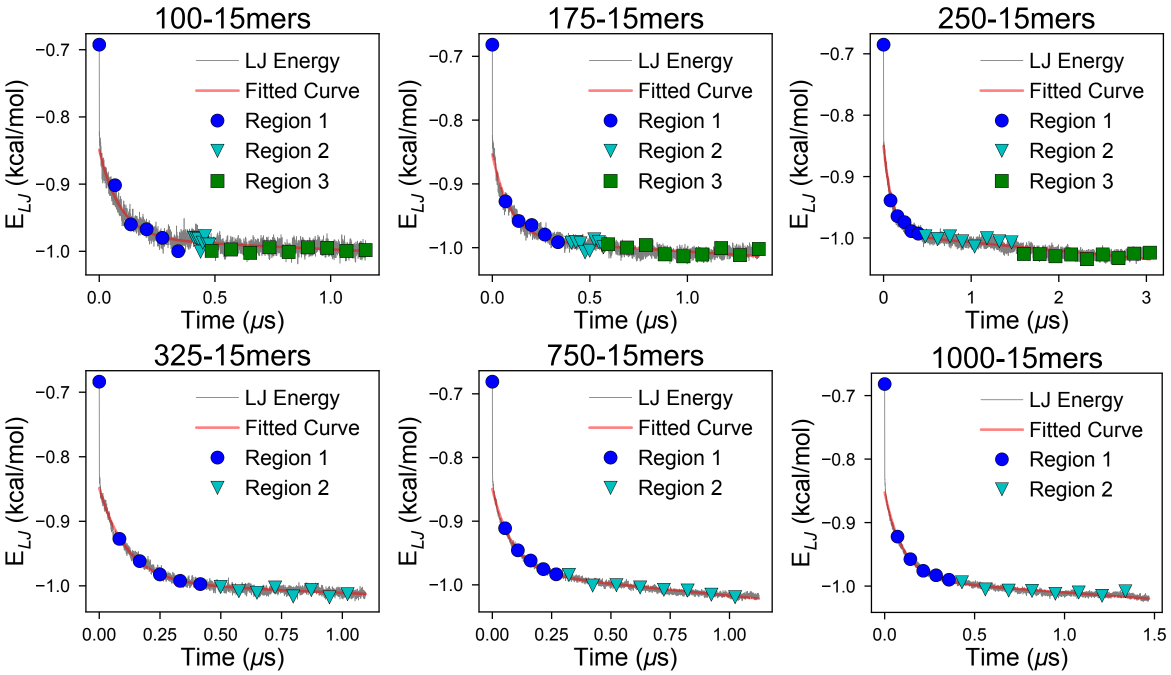

Figure S6. The per-particle Lennard-Jones potential energies for the various systems shows three regions, highlighted with blue circles, cyan triangles, and green squares for the systems with various sizes.

In this section we present our analysis of the Lennard-Jones energy to infer structural evolution. We detect three transients in the energy curve corresponding to different phases of morphology evolution: exponential decay representing formation of crystals from a disordered configuration, slow decay signifying the process of going from many small crystallites to few, highly ordered crystals and constant energy signifying equilibrium. The regions corresponding to each transient are shown in Figure S6 for simulations containing various amounts of 15mer chains. We note that not all the final energies are exactly equal. This is due to in part to systems reaching metastable states, which will require indefinite run times to relax out of. Additionally, changes in the simulation volume between the simulations can lead to unfavorable box lengths. For example, one system may be able to form a more perfect crystal when the simulation box length is commensurate with the crystal structure - resulting in a lower energy, whereas another system may never be able to form a perfect crystal because its periodicity is interrupted by the periodic boundary of the simulation volume. To quantify the duration of the exponential decrease, we conduct a least-squares fit of the per-particle Lennard-Jones energy to Equation 4, which is also used to compare the 15mer and 50mer systems. We consider a linear term in addition to an exponential term to better account for the slow transformation in Region 2, which is qualitatively linear. We calculate the derivate of Equation 4 to obtain the exponential and linear components of the slope, and identify the transition time t_s as the point at which the linear component becomes a larger than the exponential component.

To determine when equilibrium is reached and Region 2 is ended, we must first assume that the final energy measurement is in an equilibrium state (which is only a valid assumption for the systems with fewer beads, $<$ 325 15mers). We next assign the measured energies to bins containing 200 measurements each, which equates to 36 ns, and calculate the average $\bar{E}_i$ and standard deviation σ_i of each bin. We then iterate through the bins in reverse order and compare $\bar{E}_i$ and σ_i the final bin's energy $\bar{E}_{final}$ and deviation σ_{final}. When $\bar{E}_i$ is calculated to be more than the sum of the standard deviations higher than $\bar{E}_{final}$, we consider the system as not equilibrated, i.e.:

$$\text{System is} \begin{cases} \text{Not Equilibrated,} & \text{if } \bar{E}_i - \bar{E}_{final} > \sigma_i + \sigma_{final} \\ \text{Equilibrated,} & \text{otherwise.} \end{cases} \tag{5}$$

5. Small and Large Comparison for Semiordered and Disordered Systems

In the main text we consider size effect differences between the ordered large and small systems, here we expand this analysis to large and small semiordered and disordered systems (Figure S7). In the large semiordered system, some crystallites have aggregated to form alkyl-stacked lamellae (shown with colored, opaque beads), corresponding to the experimentally observed crystalline domains surrounded by an amorphous matrix (shown with gray, diffuse beads) [21]. However, in the small system, one large crystallite interspersed with smaller crystallites dominates the morphology, and the alkyl-stacked lamellae are not observed in the smaller system. The difference between these two structures is confirmed with GIXS patterns: periodic alkyl stackings are only observed in the large system. That said, the π-stacking feature along the (010) planes forms a diffuse band rather than a distinct peak - likely due to the wide range of crystallite orientations in the large morphology in the large system, whereas this feature is much stronger in the smaller system's pattern. Therefore, there is some dependence on the system size when looking at semi-ordered systems: larger systems are able to have regions of high order and amorphous regions, which are missing in smaller systems that tend to form fewer, larger crystals. This corresponds to crystallite sizes measured experimentally ($\sim$ 10-20 nm), in that the small system with a box length of $\sim$ 7 nm is smaller than the typical crystallite, whereas the larger systems with length 15 nm are the same as the average experimentally observed crystals [22]. We therefore suggest using larger system sizes ($N = 165,000$) to more accurately represent the long-range ordering characteristics of polymers.

In the case of disordered systems, both the 100 and 1000 chain systems are similar: they are comprised of many small, randomly-oriented regions of π-stacking, consisting of just a couple of chains in each case. The GIXS patterns corroborate the visual observations: there is little periodic order within the system. As such there is minimal system size dependence when the structures are more disordered; smaller simulations can be used to accurately investigate high temperatures. In summary, smaller system sizes are beneficial for fast investigations of the relationship between state-point and structure, whereas larger systems including more molecules are be needed to explore large-scale structural features.

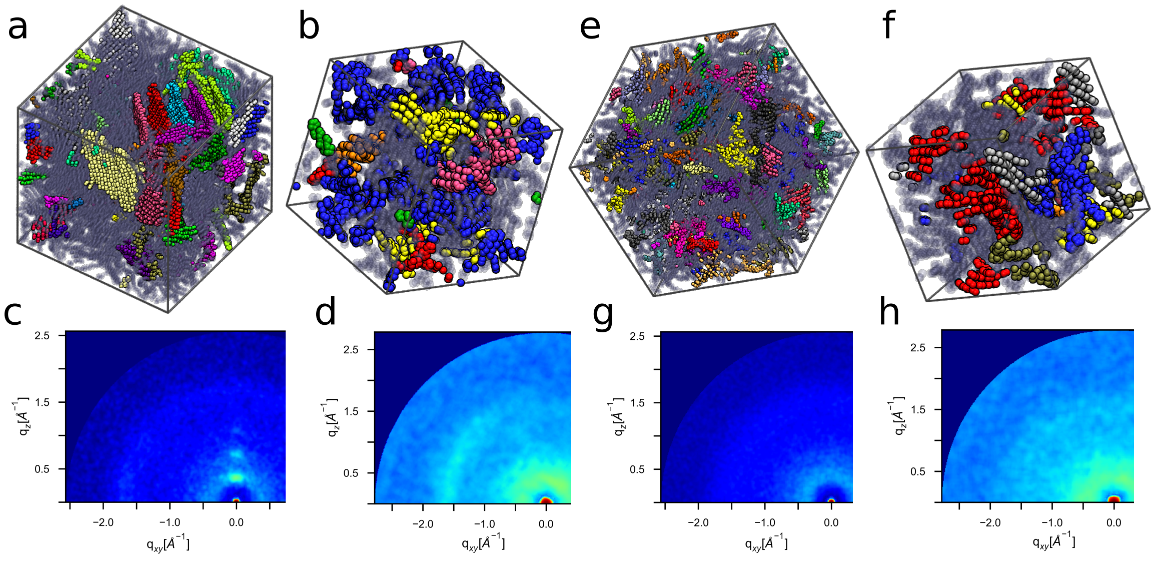

Figure S7. In cases where amorphous and structured regions exist, large volumes (a) provide more insight than their smaller counterparts (b) in that systems containing 100 15mers do not show periodic lamellae features seen in larger systems (c, d). Conversely in disordered systems, large volumes do not provide significant structural insight as compared to small volumes (e-h).

Acknowledgments: This work used the Extreme Science and Engineering Discovery Environment (XSEDE), which is supported by National Science Foundation grant number ACI-1053575 [23]. This material is based upon work supported by the National Science Foundation under Grant No. (1229709) and (1653954), and (1658076). We would like to acknowledge high-performance computing support of the R2 compute cluster (DOI: 10.18122/B2S41H) provided by Boise State University's Research Computing Department.

Author Contributions: Under the direction of Dr. Eric Jankowski, Evan Miller and Dr. Matthew Jones optimized the P3HT force-field and performed the molecular dynamic simulations. Michael Henry evaluated the MD performance on various GPUs. The code to organize and run the molecular dynamic simulations was written by Dr. Jankowski.

Conflicts of Interest: The authors declare no conflict of interest.

Abbreviations

15mer - P3HT chain containing 15 monomers
50mer - P3HT chain containing 50 monomers
AA - All-Atom
CA - Aromatic Carbon
CT - Aliphatic Carbon
ϵ - Energy Parameter
ε_s - solvent quality
GIXS - Grazing Incident X-ray Scattering
GPU - Graphical Processing Unit
HPC - High Performance Computing
m - Mass
MD - Molecular Dynamics
OPLS - Optimized Performance for Liquid Simulations
OPV - Organic Photovoltaic
P3HT - Poly(3-hexylthiophene)
ψ - order parameter
ρ - density
RDF - Radial Distribution Function
S - Sulfur
σ_{LJ} - Lennard-Jones van der Waals radius
t - Simulation Unit of Time
T - Temperature
TPS - Timesteps Per Second
UA - United-Atom

1. Huang, D.M.; Faller, R.; Do, K.; Moulé, A.J. Coarse-Grained Computer Simulations of Polymer/Fullerene Bulk Heterojunctions for Organic Photovoltaic Applications. *Journal of Chemical Theory and Computation* **2010**, *6*, 526–537. doi:10.1021/ct900496t.
2. Bhatta, R.S.; Yimer, Y.Y.; Perry, D.S.; Tsige, M. Improved Force Field for Molecular Modeling of Poly(3-hexylthiophene). *The Journal of Physical Chemistry B* **2013**, *117*, 10035–10045. doi:10.1021/jp404629a.
3. Jorgensen, W.L.; Madura, J.D.; Swenson, C.J. Optimized Intermolecular Potential Functions for Liquid Hydrocarbons. *Journal of the American Chemical Society* **1984**, *106*, 6638–6646. doi:10.1021/ja00334a030.
4. Dag, S.; Wang, L.w. Packing Structure of Poly(3-hexylthiophene) Crystal: Ab Initio and Molecular Dynamics Studies. *The Journal of Physical Chemistry B* **2010**, *114*, 5997–6000. doi:10.1021/jp1008219.
5. To, T.T.; Adams, S. Accurate Poly(3-hexylthiophene) Forcefield from First-Principle Modeling. *Nanoscience and Nanotechnology Letters* **2012**, *4*, 703–711. doi:10.1166/nnl.2012.1383.
6. LeBard, D.N.; Levine, B.G.; Mertmann, P.; Barr, S.A.; Jusufi, A.; Sanders, S.; Klein, M.L.; Panagiotopoulos, A.Z.; Barr, A.; Jusufi, A.; Sanders, S.; Klein, L. Self-assembly of coarse-grained ionic surfactants accelerated by graphics processing units. *Soft Matter* **2011**, *8*, 2385–2397. doi:10.1039/C1SM06787G.
7. Valiev, M.; Bylaska, E.J.; Govind, N.; Kowalski, K.; Straatsma, T.P.; Van Dam, H.J.; Wang, D.; Nieplocha, J.; Apra, E.; Windus, T.L.; De Jong, W.A. NWChem: A Comprehensive and Scalable Open-Source Solution for Large Scale Molecular Simulations. *Computer Physics Communications* **2010**, *181*, 1477–1489. doi:10.1016/j.cpc.2010.04.018.

8. Becke, A.D. Density-functional thermochemistry. III. The role of exact exchange. *The Journal of Chemical Physics* **1993**, *98*, 5648–5652, [z0024]. doi:10.1063/1.464913.
9. Krishnan, R.; Binkley, J.S.; Seeger, R.; Pople, J.A. Self-consistent molecular orbital methods. XX. A basis set for correlated wave functions. *The Journal of Chemical Physics* **1980**, *72*, 650–654, [arXiv:10.1063/1.438955]. doi:10.1063/1.438955.
10. Obata, S.; Shimoi, Y. Control of molecular orientations of poly(3-hexylthiophene) on self-assembled monolayers: molecular dynamics simulations. *Physical Chemistry Chemical Physics* **2013**, *15*, 9265. doi:10.1039/c3cp44150d.
11. Moreno, M.; Casalegno, M.; Raos, G.; Meille, S.V.; Po, R. Molecular modeling of crystalline alkylthiophene oligomers and polymers. *The journal of physical chemistry. B* **2010**, *114*, 1591–1602. doi:10.1021/jp9106124.
12. Borzdun, N.I.; Larin, S.V.; Falkovich, S.G.; Nazarychev, V.M.; Volgin, I.V.; Yakimansky, A.V.; Lyulin, A.V.; Negi, V.; Bobbert, P.A.; Lyulin, S.V. Molecular dynamics simulation of poly(3-hexylthiophene) helical structure In Vacuo and in amorphous polymer surrounding. *Journal of Polymer Science, Part B: Polymer Physics* **2016**, *54*, 2448–2456. doi:10.1002/polb.24236.
13. D'Avino, G.; Mothy, S.; Muccioli, L.; Zannoni, C.; Wang, L.; Cornil, J.; Beljonne, D.; Castet, F. Energetics of electron-hole separation at P3HT/PCBM heterojunctions. *Journal of Physical Chemistry C* **2013**, *117*, 12981–12990. doi:10.1021/jp402957g.
14. Yang, L.; Tan, C.H.; Hsieh, M.J.; Wang, J.; Duan, Y.; Cieplak, P.; Caldwell, J.; Kollman, P.A.; Luo, R. New-generation Amber united-atom force field. *Journal of Physical Chemistry B* **2006**, *110*, 13166–13176. doi:10.1021/jp060163v.
15. Bronstein, H.A.; Luscombe, C.K. Externally Initiated Regioregular P3HT with Controlled Molecular Weight and Narrow Polydispersity. *Journal of the American Chemical Society* **2009**, *131*, 12894–12895, [arXiv:1408.1149]. doi:10.1021/ja9054977.
16. Koch, F.P.V.; Smith, P.; Heeney, M. "Fibonacci's Route" to Regioregular Oligo(3-hexylthiophene)s. *Journal of the American Chemical Society* **2013**, *135*, 13695–13698. doi:10.1021/ja4057932.
17. Grubbs, R.B.; Grubbs, R.H. 50th Anniversary Perspective : Living Polymerization—Emphasizing the Molecule in Macromolecules. *Macromolecules* **2017**, *50*, 6979–6997. doi:10.1021/acs.macromol.7b01440.
18. Ihn, K.J.; Moulton, J.; Smith, P. Whiskers of poly(3-alkylthiophene)s. *Journal of Polymer Science Part B: Polymer Physics* **1993**, *31*, 735–742. doi:10.1002/polb.1993.090310614.
19. Mena-Osteritz, E.; Meyer, A.; Langeveld-Voss, B.M.W.; Janssen, R.A.J.; Meijer, E.W.; Bäuerle, P. Two-Dimensional Crystals of Poly(3-Alkyl- thiophene)s: Direct Visualization of Polymer Folds in Submolecular Resolution. *Angewandte Chemie International Edition* **2000**, *39*, 2679–2684. doi:10.1002/1521-3773(20000804)39:15<2679::AID-ANIE2679>3.0.CO;2-2.
20. Brinkmann, M.; Wittmann, J.C. Orientation of regioregular poly(3-hexylthiophene) by directional solidification: A simple method to reveal the semicrystalline structure of a conjugated polymer. *Advanced Materials* **2006**, *18*, 860–863. doi:10.1002/adma.200501838.
21. Brinkmann, M.; Rannou, P. Effect of Molecular Weight on the Structure and Morphology of Oriented Thin Films of Regioregular Poly(3-hexylthiophene) Grown by Directional Epitaxial Solidification. *Advanced Functional Materials* **2007**, *17*, 101–108. doi:10.1002/adfm.200600673.
22. Liu, F.; Chen, D.; Wang, C.; Luo, K.; Gu, W.; Briseno, A.L.; Hsu, J.W.; Russell, T.P. Molecular weight dependence of the morphology in P3HT:PCBM solar cells. *ACS Applied Materials and Interfaces* **2014**, *6*, 19876–19887. doi:10.1021/am505283k.
23. Towns, J.; Cockerill, T.; Dahan, M.; Foster, I.; Gaither, K.; Grimshaw, A.; Hazlewood, V.; Lathrop, S.; Lifka, D.; Peterson, G.D.; Roskies, R.; Scott, J.R.; Wilkens-Diehr, N. XSEDE: Accelerating Scientific Discovery. *Computing in Science & Engineering* **2014**, *16*, 62–74. doi:10.1109/MCSE.2014.80.
